# Hall’s Journal for 1876

**Published:** 1876-01

**Authors:** 


HALL'S

### JOURNAL OF HEALTH.

Vol. XXIII—JANUARY, 1876—No. 1. Whole No. 265.

### HALL'S JOURNAL FOR 1876.

We enter upon the twenty-third year of our own existence, and the one hundredth of the life of the Republic, with a very decided determination to make our old and favorite magazine as entertaining and valuable as possible during the coming twelve months. We have never been profuse in promises, but have constantly endeavored to supply good advice and salutary hints to those who have looked to us for assistance in right living. We have said, times without number, that a sensible man or woman having access to suitable health literature, and living wisely, can reduce the average annual illness to the merest trifle; in fact to the unavoidable accidents of life; and it is our province to supply the needful hints which are sufficient in themselves to carry a family through the vicissitudes of most diseases which are the common lot of humanity.

But we have a still loftier aim than that of imparting advice in hours of suffering. What we hold is that not one thousandth part of the disease and misery which afflict humanity need exist, if the world would eat and drink, and dress and bathe, and exercise and breathe, wisely. So far as our influence extends, we are imparting such instruction as our long experience and close observation have told us will be effectual in warding off disease. That our advice is followed, we know; that the result is to lessen human suffering, we have abundant testimony. This assurance, coming as it does from multitudes of readers, encourages us to continue in our work of educating the masses in the direction of a higher, more careful, more temperate, and more watchful life.

We look now for a year of good results. We have never seen the time when the desire for knowledge concerning these bodies of ours, their demands and needs, was as general and as earnest as to-day. Especially is this true in the matter of foods. People who have heretofore scarcely stopped to ask whether this or that article was strengthening or hurtful, now inquire earnestly if they are really eating for strength and health, or for weakness and misery. This tendency to inquiry is very gratifying to such as have long labored almost alone in this important field.

We have yet a number of gigantic evils to vanquish, and we hope to deal them some heavy blows during the coming year. In this work we are grateful for the assistance of our friends. We discover an almost universal readiness on the part of our old subscribers to renew their subscriptions, and we must do them the justice to say that their influence has brought us a goodly number of new subscribers within the last sixty days. It takes but few words from one of our friends to a neighbor to secure for us another constant reader. Whenever a friend is willing to devote the necessary time to a thorough canvass of his neighborhood in behalf of the JOURNAL, we are always glad to liberally compensate him for the service. Thousands of persons who are now without employment could make an excellent living and accomplish a great deal of good, by soliciting subscribers for our magazine. It is pleasant work, for every body in America knows something about HALL'S JOURNAL OF HEALTH. If they never saw a copy of it, they have seen extracts from its writings; for nearly every newspaper in the land finds from week to week many little nooks into which our choice paragraphs neatly fit. The domestic and health-columns of nine-tenths of the papers in the country have long looked to our pages for a supply of useful reading; and hardly a day passes in which we do not find our articles reproduced, often, too, without one word of reference to their source. This has long been the case, and we have never complained unless our writings have been distorted and abused. We copyright our JOURNAL entire; but we give the fullest permission for all publishers to use the contents of this Magazine in a fair and courteous manner. We do not, however, allow our writings to be wrongfully used, or our title to be placed upon other periodicals. This has been done by some very wicked persons, and we have suffered greatly by their dishonesty, as have those whose money, intended for us, has accidentally fallen into the hands of these bad men. In future all publications employing such portion of our title as would amount to a deception, will be legally suppressed, and judgment will be secured for ample damages.

We conclude with the expression of the hope that the year 1876 will manifest a renewal of business activity; that the lessons of the late years of depression will not have been conned in vain, and that the thousands who have been compelled to practice a rigid economy of late, on account of the great shrinkage in values, will find that the discipline has not been without some good result. In the hope of better and brighter days for our friends, we wish one and all a glad and prosperous and happy New Year!

